# Towards a dry separation method of radioscandium from bulk amounts of titanium

**DOI:** 10.1007/s10967-025-10092-4

**Published:** 2025-04-19

**Authors:** Xiuyun Chai, Mohamed F. Nawar, Matthias Lüthi, Ronald Zingg, Christian Kottler, Andreas Türler

**Affiliations:** 1 Department of Chemistry, Biochemistry, and Pharmaceutical Sciences, University of Bern, 3012 Bern, Switzerland; 2 Federal Institute of Metrology, METAS, 3003 Bern-Wabern, Switzerland

**Keywords:** Theranostics, ^47^Sc, Dry separation method, Titanium targets

## Abstract

True theranostic radionuclide pairs are gaining interest in nuclear medicine. However, their application is limited due to their reduced availability. ^47^Sc is a promising therapeutic partner to the diagnostic ^43^Sc and ^44^Sc due to its ideal half-life and beta energy. This study attempted to develop a dry, gas–solid phase separation of non-carrier-added radioscandium from macro amounts of titanium. The results showed a 99.9% release of ^47^Sc from Ti foil of 50 μm thickness at 1100 °C in vacuum. Furthermore, a Monte Carlo model was developed to describe the release of Sc from Ti as a function of temperature and foil thickness. With this model the desorption energy of Sc from Ti surfaces was determined to be 390 ± 5 kJ/mol in excellent agreement with theoretical predictions. In an attempt to recover the evaporated ^47^Sc on a cold finger, highest overall yields of 65% were observed for gold surfaces. For other catcher materials such as Ti, Ta, and brass significantly lower overall yields between 27 and 47% were determined. Furthermore, it was not possible to quantitatively remove the ^47^Sc from the Au catcher, even when washing with concentrated acids. Attempts to increase the release of scandium from thick, sintered Ti samples failed to show an improvement over solid titanium.

## Introduction

Recently, precision medicine has attracted considerable interest in nuclear medicine applications. It provides tailored diagnosis and therapy characteristics for specific patients to enhance the efficacy of medical care services [1, 2]. In this regard, radionuclides of Sc, namely ^43^Sc and ^44^Sc as positron emitters and ^47^Sc as beta emitter, have gained growing attention. On the one hand, ^43,44^Sc are suitable for imaging with relatively short half-lives (^43^Sc: *t*_*1/2*_ = 3.891 h, ^44^Sc: *t*_*1/2*_ = 3.97 h) and low-energy positron energies (^43^Sc: E_avg__β_^+^ = 476 keV, ^44^Sc: E_avg__β_^+^ = 632 keV) [3–5]. On the other hand, ^47^Sc has a 3.349 d half-life and decays by 100% _β_^−^ emission with adequate energy (E_avg__β_^−^ = 162 keV), allowing for an ideal therapeutic partner, which is a potential candidate for the theranostic treatment of metastatic cancers. Furthermore, ^47^Sc emits a low-energy γ-ray (E_γ_ = 159.381 keV), enabling the visualization of the therapeutic dose through SPECT imaging [6–9].

In addition, regarding its valence state, hardness, or ionic radius, the trivalent ions of scandium confer unique chemical characteristics that mimic lanthanide-like properties—for instance, ^177^Lu. The production of ^177^Lu has some critical challenges that should not be overlooked. For example, its production requires high neutron flux reactors [10]. In addition, the separation of ^177^Lu from macro amounts of the irradiated ytterbium target necessitates a tedious radiochemical separation and purification routine. Furthermore, this approach is expensive, as it requires the use of enriched ^176^Yb targets [10, 11].

^47^Sc can be feasibly produced by an electron accelerator. The possible photonuclear reactions are ^48^Ti(γ,p)^47^Sc, ^49^Ti(γ,pn)^47^Sc, ^50^Ti(γ,p2n)^47^Sc, or ^51^V(γ,α)^47^Sc. The ^48^Ti(γ, p)^47^Sc reaction is the most relevant photonuclear reaction because of the high abundance of ^48^Ti, as it is the most abundant isotope occurring in nature [12, 13]. In a previous study, the photonuclear production of non-carrier added (nca) ^47^Sc from natural Ti foils and its subsequent chemical processing and purification were developed [14]. Rotsch et al. eliminated the unwanted contaminations of ^46^Sc and ^48^Sc by using enriched ^48^Ti targets [15]. Moreover, the relatively lower radioactive waste and lower cost also appear to be advantageous compared to the production of radionuclides in nuclear reactors [16].

Various methods have been explored for the separation of ^47^Sc from irradiated targets [17]. Typically, wet separation procedures were widely used. These methods involve the acid dissolution of the target material, followed by ion exchange, solvent extraction, and extraction chromatography, necessitating harsh reaction conditions. For instance, they involve the use of highly potent acids and peroxidation chemicals (e.g., HF (48%), HCl, and H_2_O_2_) [14]. As an alternative approach, Wittwer et al. developed a gas phase separation method, wherein Ti foils were heated in vacuum between 900 and 1500 °C, resulting in the evaporation of ^47^Sc [18].

Our study aims to evaluate the applicability of separating the produced radioscandium isotopes in nca quality from macro amounts of irradiated natural Ti targets. Since ^47^Sc is the predominant radioactivity produced in photonuclear reactions on natural Ti, only the behaviour of this isotope was investigated. For production of ^47^Sc in quantity and quality for medical applications, the use of highly enriched ^48^Ti would be required.

We comprehensively analyzed several factors influencing the release of ^47^Sc from irradiated Ti foils such as temperature and foil thickness. Moreover, our investigation encompasses an assessment of different catcher materials, focusing on identifying the most promising material for recovering the released ^47^Sc.

## Experimental

### Materials

All chemicals were of analytical grade purity and were used without further purification. Milli-Q water was used for the preparation of solutions and washings. Nitric acid and hydrochloric acid were purchased from Merck, Darmstadt, Germany. Titanium foils (99.94% and 99.999% purity) with different thicknesses (0.025, 0.05, 0.1, 0.25, 0.500, and 1 mm) were purchased from Goodfellow, Huntingdon PE29 6WR, England. Titanium sponge (99.9% purity, particle size of 3 mm), Ti powder (99.5% purity, particle size of 0.045 mm), Ti dihydride (99.5% purity, particle size of 0.15 mm), tantalum foil (99.999% purity, thickness of 0.025 mm), and gold foil (99.999% purity, thickness of 0.25 mm) were purchased from Goodfellow, Huntingdon PE29 6WR, England. A brass foil CuZn37 (thickness of 0.2 mm) was purchased from Metall Service Menziken, Menziken, Switzerland.

### Instrumentation

Radiometric identifications and standard measurements were carried out by using a multichannel analyser (MCA), Canberra Series, Mirion Technologies, Inc., Meriden, CT, USA, coupled with a high-purity germanium detector (HPGe). Samples were counted at a low dead time (< 5%). The radioactivity levels were determined by quantifying the 159.4 keV photo peaks corresponding to ^47^Sc. A thermostat water bath (Lauda GmbH, Lauda-Königshofen, Germany) was used for cooling the separation system. A vacuum of 1 × 10^−3^ Pa was maintained during our investigation using a HiCube vacuum pump (Pfeiffer, Aßlar, Germany). The target materials were pressed using a Maassen Pellet Press MP150 (Maassen GmbH, Reutlingen, Germany). Different temperature values were studied using a tube furnace from Eurotherm, Limburg an der Lahn, Germany.

### Production of ^47^Sc

^47^Sc was produced by the photonuclear reaction ^48^Ti(γ,p)^47^Sc. Titanium targets were irradiated with Bremsstrahlung created by the M22 Microtron from Scanditronics at the Federal Institute of Metrology METAS, Bern-Wabern, Switzerland. Electrons, accelerated to an energy of 21 MeV were used to produce Bremsstrahlung in a Au converter target. The pulsed electron beam has a peak current of approximately 30 mA and a pulse duration of 3 μs. The repetition rate was 150–200 Hz, and the irradiation time for one target was between 5 to 7 h.

### Effect of temperature on the release of ^47^Sc from Ti

**Fig. 1:**
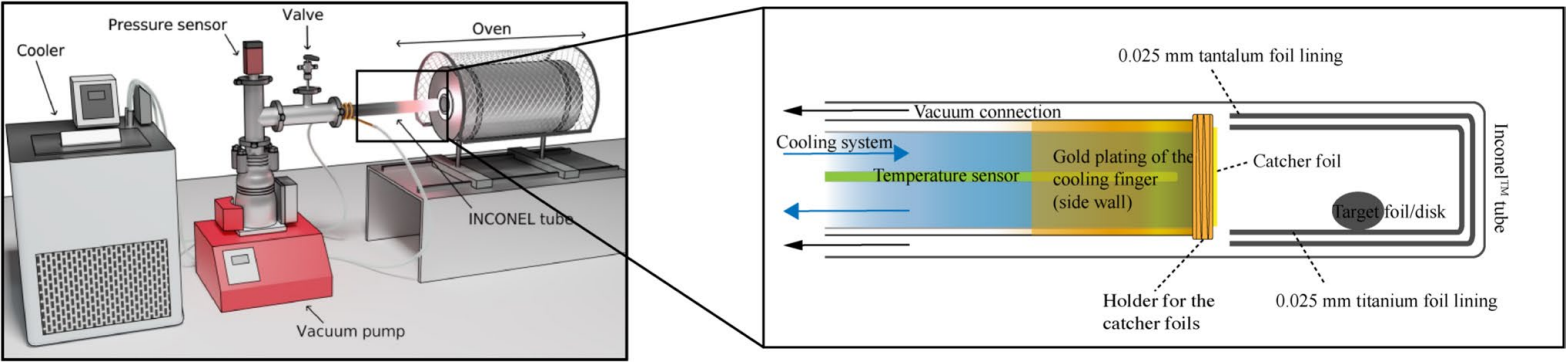
Schematic illustration of the separation equipment and a representation of the inner vacuum release setup

The irradiated Ti targets (0.025 mm, solid foil) were placed inside an Inconel™ steel tube of 2 cm inner diameter completely lined with tantalum and titanium foils. The assembly is placed in a tube furnace that can be heated to different temperatures and connected to a turbomolecular vacuum pump. A cooling finger was constructed to facilitate the recovery of the released ^47^Sc from the irradiated targets. The cooling system consists of a cooling finger placed inside the Inconel™ tube and directly attached to the vacuum pump. The cooling water was allowed to circulate through the cooling finger and outer part of the Inconel™ tube. The cooling water was kept at a constant temperature of 5–20 °C with a thermostat. The complete setup is shown in Fig. 1.

The irradiated targets were heated to 300 °C under a vacuum of 1 × 10^−3^ Pa for 1 h to eliminate traces of water and oxygen. This step prevented the oxidation of the irradiated Ti target surface before the release of ^47^Sc. Subsequently, the oven temperature was increased to several temperature values (900, 950, 1000, 1050, and 1100 °C). Each value was maintained under a vacuum of 1 × 10^−3^ Pa for 2 h.

Eventually, at the end of the separation process, the entire system was allowed to cool before removing the irradiated target.

### Effect of target thickness on the release of ^47^Sc from Ti

The influence of the target thickness on the release of ^47^Sc was evaluated. A wide range of target thicknesses (0.025, 0.05, 0.1, 0.25, 0.5, and 1 mm) was selected. During our investigations, a constant reaction time of 2 h, a reaction temperature of 1100 °C, and a vacuum of 1 × 10^−3^ Pa were applied. Each sample was placed in the middle of the Inconel™ tube as shown in Fig. 1 right (zoom in part).

### Effect of different catcher materials on the recovery of ^47^Sc

In order to recover the released ^47^Sc from the irradiated Ti targets, the sorption properties of different catcher foils were evaluated. These recovery foils include titanium, brass, tantalum, and gold. Each foil was individually attached to the head of the cooling finger. Subsequently, it was allowed to cool down and was measured using HPGe γ-ray spectrometry. Finally, the total recovery ratio of ^47^Sc was evaluated.

### Effect of target material and composition on the release of ^47^Sc from Ti

The change in the release of radioscandium from different target materials was studied. These materials are Ti foil, Ti sponge, sintered mixtures of Ti and Ti hydride powders. All targets with a thickness of 1 mm were subjected individually to 1100 °C for 2 h. The sintered targets were prepared by mixing Ti and TiH_2_ powders in ratios of 1:1 and 2:3. Then, each mixture was pressed and directly heated up to 1050 °C under a vacuum of 1 × 10^−3^ Pa for 1 h. Eventually, the sintered targets were cooled to room temperature overnight.

## Theory

The release and the recovery ratios of ^47^Sc from irradiated Ti targets were calculated according to the following formulas:

1 $$ \begin{array}{*{20}c} {R_{rel} = \frac{{C_{i} - C_{f} }}{{C_{i} }} \times 100} \\ \end{array} $$

2 $$ \begin{array}{*{20}c} {R_{rec} = \frac{{C_{a} }}{{C_{i} - C_{f} }} \times 100} \\ \end{array} $$

where *R*_*rel*_ and *R*_*rec*_ are the relative release and the relative recovery ratio, while C_i_, C_f_, and C_a_ are the measured initial, residual, and catcher foil radioactivity expressed in Becquerel (Bq).

### Monte carlo modeling

The release of Sc from irradiated Ti can be described as a two step process. In a first step the homogenously distributed Sc atoms have to diffuse to the surface of the Ti matrix. In a second step, the Sc atoms are evaporated from the Ti surface into vacuum. While the first step is governed by the experimental parameters (foil thickness, experiment duration) and the temperature dependent diffusion coefficient, the second step is governed by the parameters experiment duration, temperature and the adsorption enthalpy of Sc on the Ti surface. This quantity has not been measured but can be extracted from the experimental data and compared to model predictions.

Under the assumption that radioscandium is produced homogenously in the irradiated Ti disk in photonuclear reactions at the tracer level and that the thickness of the Ti disks is much smaller than their diameter, the rate of Sc atoms reaching the Ti surface by diffusion can be described by the expression:

3 $$ \begin{array}{*{20}c} {F\left( {D\left( T \right), t,d} \right) = 1 - \frac{8}{{\pi^{2} }}\exp \left( { - \frac{D\left( T \right)t}{{d^{2} }}} \right)} \\ \end{array} $$

where *D(T)* is the temperature dependent diffusion coefficient (cm^2^/s), *T* is the temperature (K), t is the experiment duration/heating time (s), and d is the target foil thickness (cm) [18, 19]. Temperature dependent diffusion coefficients of Sc in Ti have been dertermined experimentally by Askill and Gibbs [20] in the relevant temperature range and can be expressed by

4 $$ D\left( T \right) = 10^{{\left( {0.0039 \times T - 13.252} \right)}} \left( {cm^{2} /s} \right) $$

by fitting the available data (*T* in K).

Assuming a reversible simple adsorption/desorption process for the release of Sc from the Ti surface into vacuum, the mean adsorption residence time is governed by the adsorption enthalpy of Sc on the Ti surface expressed by

5 $$ \overline{\tau }_{a} = \tau_{0} \cdot e^{{{\raise0.7ex\hbox{${ - \Delta H_{a} }$} \!\mathord{\left/ {\vphantom {{ - \Delta H_{a} } {RT}}}\right.\kern-0pt} \!\lower0.7ex\hbox{${RT}$}}}} \left( s \right) $$

where $\overline{{\tau }_{a}}$ is the mean adsorption residence time, τ_0_ is the period of oscillations of the Sc atom in the adsorbed state perpendicular to the surface (taken as 1.6 × 10^–13^ s in the Lindemann formula), − Δ*H*_a_ is the adsorption enthalpy in kJ/mol, R the gas constant, and T the temperature in K. An exponential probability density distribution holds for $\overline{{\tau }_{a}}$ [21].

The two step process of diffusion of Sc to the Ti surface and its evaporation into vacuum was modeled using a Monte Carlo approach. The function *F(D(T),t*_*exp*_*,d)* describes the probability of a Sc atom to reach the Ti surface during an experiment of length *t*_exp_ at a given temperature and foil thickness. The individual history of a Sc atom was simulated by selecting a random time point *t* within the experiment duration and checking if the Sc atom had already diffused to the surface (Eq. 3). Since the probability of a Sc atom to reach the surface is lower for early time points compared to later time points during the experiment, the random time *t* was selected in a manner to reflect the probability density distribution generated by *F(D(T),t,d).* As experimental parameters the temperature *T*, the thickness of the foil *d* and the diffusion coefficient according to Eq. 4 were used. If the Sc atom had not reached the surface, the history of a new Sc atom was modeled and the number of Sc atoms that did not evaporate from the Ti was increased by one. Once a Sc atom had reached the surface an adsorption residence time for a selected − Δ*H*_a_-value was generated and added to the time point at which the Sc atom had reached the surface. If this combined time was shorter than the experiment duration the Sc atom was registered as having evaporated into vacuum, whereas if the combined diffusion and residence time was longer than the experiment time the Sc atom was registered as still being adsorbed to the Ti surface. For a constant foil thickness and − Δ*H*_a_-value, the history of 10′000 Sc atoms was simulated in the temperature range between 850 and 1150 °C in steps of 25 °C for − Δ*H*_a_-values between − 385 and − 395 kJ/mol in steps of 1 kJ/mol and the resulting relative release of Sc, *R*_*rel*_, was compared with experimental results. The position of the resulting sigmoid curve of *R*_*rel*_ as a function of temperature reacts very sensitively to changes of the − Δ*H*_a_-value and, therefore, the adsorption enthalpy of Sc on a Ti surface can be extracted from the experimental data by e.g. a least squares fitting procedure.

## Results and discussion

### Effect of temperature on the release of ^47^Sc from Ti

**Fig. 2:**
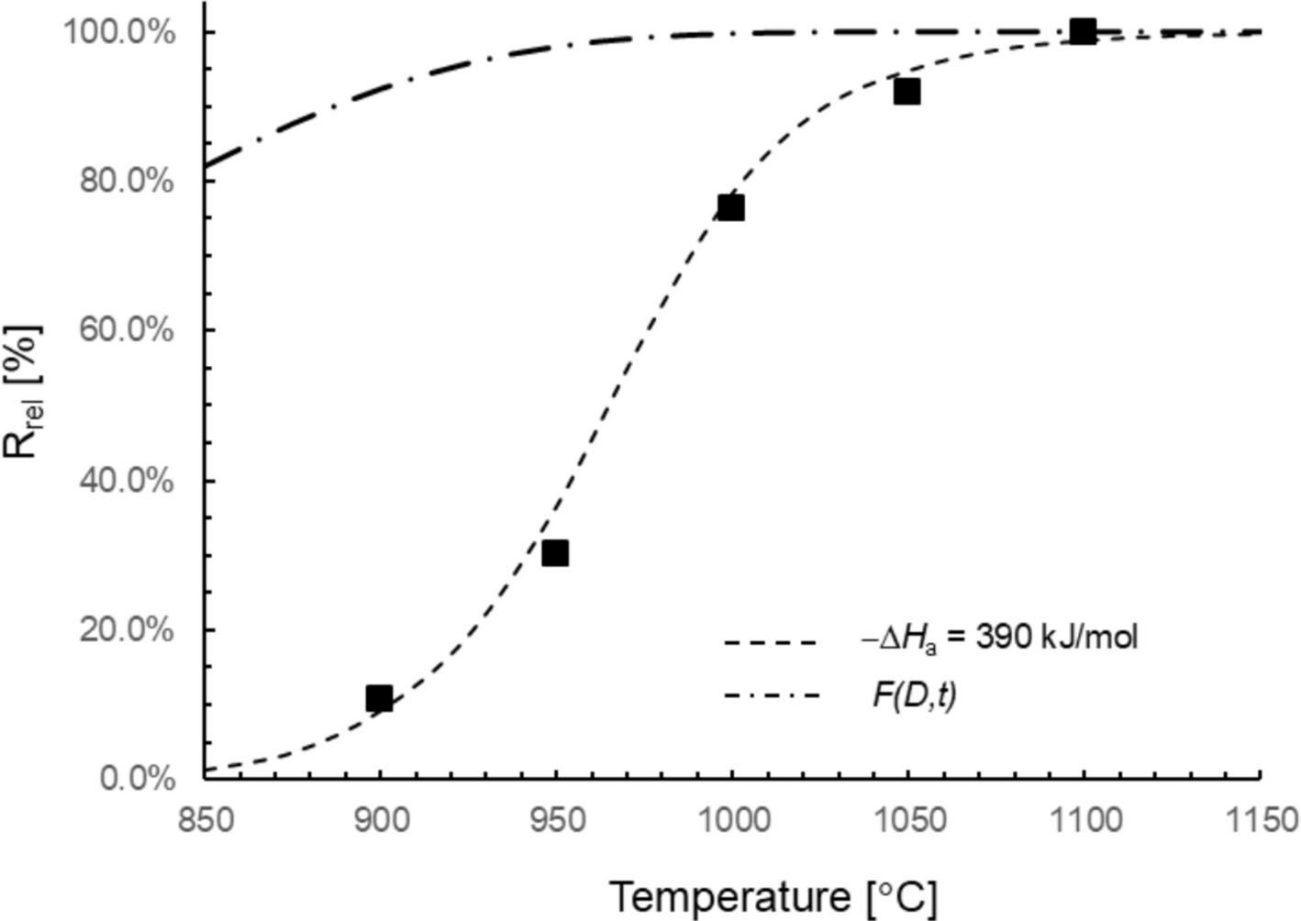
Influence of temperature on the release of ^47^Sc from Ti foils with a thickness of 0.025 mm. The dashed line shows the result of a Monte Carlo model (see theory section) simulating the diffusion and evaporation of Sc in Ti with an adsorption enthalpy of -390 kJ/mol. The dash dotted line shows the calculated probability of a Sc atom to reach the Ti surface *F(D,t)* (Eq. 3)

Release experiments of ^47^Sc from Ti were conducted in a temperature range from 900 to 1100 °C to determine the optimum temperature value that shows the maximum release of ^47^Sc from the irradiated Ti targets. Figure 2 illustrates the release of ^47^Sc from Ti targets with a thickness of 0.025 mm at different temperatures. In our experiments uncertainties apart from counting statistics, which were of the order 0.1–0.2%, were estimated to be of the order of 1–2%. Therefore, the size of the data points in Fig. 2 is larger than the associated uncertainties. The data presented in this figure show that the release of ^47^Sc notably increased with increasing temperature, which agrees with previously published studies [18, 20]. The maximum release of 99.9% was observed at 1100 °C. A former study achieved a 97.4 ± 0.12% release of ^47^Sc when the temperature was raised to 1400 °C. Compared to this study, our findings demonstrate a relatively higher release of ^47^Sc at a lower reaction temperature. Consequently, according to the obtained results, we selected a temperature of 1100 °C for subsequent investigations.

Using a two step Monte Carlo model of diffusion and evaporation of Sc from Ti, as discussed in the theory section, the history of 10′000 Sc atoms was modeled and the release ratio was determined for each temperature setting for − Δ*H*_a_-values between 385 kJ/mol and 395 kJ/mol. The best fit with the experimental data was obtained for − Δ*H*_a_ = 390 ± 5 kJ/mol (dashed line in Fig. 2). Furthermore, the model accurately describes the shape of the release of Sc from Ti as a function of temperature. Also shown is the probability of a Sc atom to reach the Ti surface as calculated with Eq. 3 (dash dotted line). Evidently, the evaporation process of Sc from the Ti surface is the dominating process for temperatures below 1100 °C, as Eq. 3 clearly fails to describe the observed behavior.

Since there is no experimental data for the adsorption enthalpy of Sc on Ti surfaces, the obtained value must be compared with model predictions. In [22], using the Eichler-Miedema model, a desorption enthalpy of Sc on Ti surfaces of 386 kJ/mol was predicted in excellent agreement with our experimental value.

### Effect of target thickness on the release of ^47^Sc from Ti

**Fig. 3:**
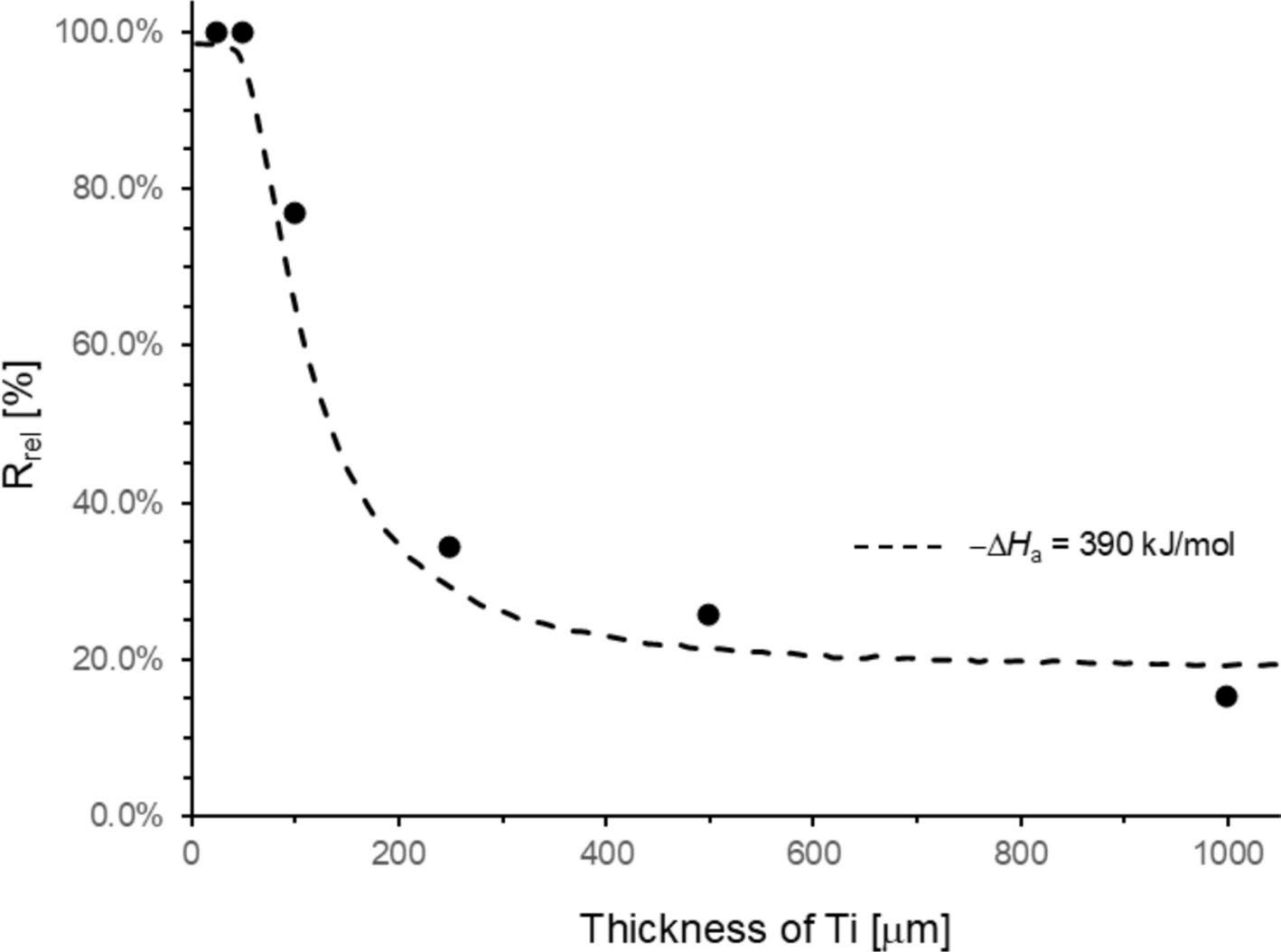
Influence of Ti target thickness on the release of ^47^Sc at 1100 °C and 2 h experiment time. The dashed line shows the result of a Monte Carlo model (see theory section) simulating the diffusion and evaporation of Sc in Ti with an adsorption enthalpy of -390 kJ/mol

The effect of Ti target thickness on the release of ^47^Sc was investigated. The release temperature was adjusted to 1100 °C, and the experiment time was 2 h. The results are shown in Fig. 3. Great care was taken to measure the foils in the same counting geometry before and after the release experiments. The results show that the maximum ^47^Sc release of 99.9 ± 0.15% was achieved at a target thickness of 0.025 and 0.05 mm. The uncertainties given are 1 sigma uncertainties containing purely counting statistics. A crucial measurement at 0.100 mm thickness was repeated twice, the results were 76.81 ± 0.10% and 78.63 ± 0.11%. So, it appears that in our experiments other sources of uncertainty apart from counting statistics were of the order of 1–2%. By increasing the target thickness from 0.25 mm to 1.00 mm, the release decreased from 34.36 ± 0.07% to 15.15 ± 0.05%.

Again, the Monte Carlo model with an adsorption enthalphy of − Δ*H*_a_ = 390 kJ/mol was applied and compared to the experimental data. Here, the probability of a Sc atom to reach the Ti surface (Eq. 3) is the dominating factor, while the evaporation step at 1100 °C introduces only a correction of few % at thin Ti target thicknesses. For larger thicknesses (> 200 μm) the correction is negligible. An even better fit to the experimental data could be achieved by adjusting the value of the diffusion coefficient. However, the values would no longer be compatible with the data of Askill and Gibbs [20].

### Effect of different catcher materials on the recovery of ^47^Sc

In order to recover the released ^47^Sc from the Ti targets, a series of catcher foils were used, such as Ti, Ta, brass, and Au. The foils were attached to the head of the cooling finger. The recovery capacity of each foil was tested separately. After each investigation, the catcher foil was measured using a HPGe γ-ray spectrometer, and its recovery ratio was calculated according to Eq. (2).

Table 1 illustrates different catcher foils and their respective recovery capacities. Under our experimental conditions, maximum recovery capacities of 65% and 47% were reached using Au and brass foils, respectively. In addition, Ti and Ta foils displayed similar recovery capacities of approximately 30%. The obtained results reveal that out of the catcher foils investigated in this study, Au foil exhibited the best recovery profile for ^47^Sc. However, subsequent experiments to wash the recovered Sc off the Au foil with dilute or concentrated acids failed.

**Table 1:** Influence of different catcher foils on the recovery of evaporated ^47^Sc

Catcher foil	Thickness (mm)	Diameter (mm)	R_rec_ (%)
Ti foil	0.025	25	30
Brass foil	0.100	25	47
Au foil	0.250	25	65
Ta foil	0.100	25	27

In order to understand these results the adsorption enthalpy of Sc on Au was estimated [23] and compared to the value for the adsorption of Sc on Ti. Using Eq. 4 of [23] − Δ*H*_a_(Sc/Au) = 370 ± 23 kJ/mol was calculated using a sublimation enthalpy of Sc of 332.7 kJ/mol, not much different from − Δ*H*_a_(Sc/Ti) = 390 ± 5 kJ/mol for Ti determined in this work. The significant difference in recovery of Sc by Au and Ti can probably be attributed to the fact, that Sc is quickly migrating/dissolving into the Au matrix and a resublimation is excluded, while this is not the case for Ti.

### Effect of target material composition on the release of ^47^Sc from Ti

It was speculated that a highly porous Ti target might positively influence the release of ^47^Sc and, therefore, allow the use of thicker Ti targets. Herein, we studied different materials, such as Ti foil, Ti sponge, and sintered mixtures of Ti and Ti hydride powders in ratios of 1:1 and 2:3 with the same thickness at a release temperature of 1100 °C. The results are displayed in Table 2. The table shows that the highest ^47^Sc release was observed from Ti foil with a release of 15.15 ± 0.05%. This value is about twice higher than a Ti sponge target with the same thickness. Furthermore, the ^47^Sc releases from the sintered mixtures of Ti and TiH_2_ powders in ratios of 1:1 and 2:3 were 12.12 ± 0.08% and 10.05 ± 0.13%, respectively. As a measure of porosity, the density of the materials was determined. The results for solid Ti and sintered TiH_2_ were in agreement with literature data [24]. These findings demonstrate that the used Ti materials did not exhibit a highly porous morphology and did not improve the release of ^47^Sc.

- The materials were pressed as a disk target.
- The sintering procedure was performed as described in Sect. 7 of the experimental part.

**Table 2:** Influence of target material composition on the released ^47^Sc

Target composition	Reaction temperature (°C)	Target thickness (mm)	^47^Sc released (%)	Density (g/cm^3^)
Ti foil	1100	1	15.15 ± 0.05	4.676
Sintered Ti:TiH_2_ (1:1)^a,b^	1100	1	12.12 ± 0.08	3.732
Sintered Ti:TiH_2_ (2:3)^a,b^	1100	1	10.05 ± 0.13	3.748
Ti Sponge granules^a,b^	1100	1	8.10 ± 0.12	4.529

a) The materials were pressed as a disk target.

b) The sintering procedure was performed as described in Sect. 7 of the experimental part

**Fig. 4:**
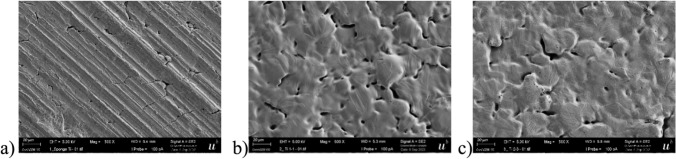
**a** Pressed Ti sponge granules, **b** sintered Ti/TiH_2_ 1:1 **c** sintered Ti/TiH_2_ 2:3 SEM images at 500 × magnification

In order to better understand the relatively poor release of Sc from sintered Ti, the morphology of the materials was analyzed using scanning electron microscopy (SEM). The corresponding pictures with 500 × magnification are shown in Figs. 4a-c.

As can be seen from Figs. 4a-c, all materials are quite homogenous and not as expected individual granules attached to each other with significant hollow structures. This might explain why sintered materials and pressed Ti sponge did not show an improved Sc release. Rather, the hollow structures might even trap some of the released Sc, leading to a reduced Sc release.

## Summary and conclusions

Our main objective was to investigate the optimum separation conditions of radioscandium from bulk amounts of irradiated Ti targets using a thermal release procedure. The experimental parameters of temperature, target thickness, and target material were systematically evaluated. According to the results, the highest separation ratio took place at a temperature of 1100 °C and foil thicknesses of ≤ 50 μm. However, the fraction of released radioscandium decreased significantly with increasing Ti thickness, the limiting factor being the diffusion of Sc to the Ti surface. Unfortunately, due to experimental constraints, the temperature could not be increased significantly. It was speculated that Ti sponge or sintered Ti would be thin enough to allow a quick diffusion of Sc to the Ti surface while exhibiting sufficient porosity for a quick release of Sc into vacuum. Our investigations showed that the investigated materials did not improve the release of Sc, which could be explained by the rather homogenous appearance of the surfaces using scanning electron microscopy (SEM). Our study investigated the capacity of different materials to recover ^47^Sc released during the separation process. Au foil showed a promising capacity as a catcher material to recover the released ^47^Sc, but the recovered Sc failed to wash off the Au foil even with concentrated acids. A possible alternative to be tested might be the possibility to process foils with ≤ 50 μm at only 850 to 900 °C, where still a substantial fraction of Sc should reach the Ti surface without evaporating and to dissolve the thus separated Sc from the Ti surface for further processing. In conclusion, the thermal release of Sc from thin Ti foils was shown to be very efficient and would warrant the irradiation of stacks of thin Ti foils (≤ 100 μm). Using the Monte Carlo model developed in this work and associated thermochemical data for the release of Sc from Ti surfaces will enable an efficient design of future technological developments. However, the efficient recovery of the evaporated Sc proved to be difficult. Therefore, further investigations are required to establish the dry separation method of radioscandium from bulk amounts of Ti as a viable option for the future production of nca ^47^Sc.
